# Patient coping strategies in COPD across disease severity and quality of life: a qualitative study

**DOI:** 10.1038/npjpcrm.2016.51

**Published:** 2016-09-15

**Authors:** Sarah B Brien, George T Lewith, Mike Thomas

**Affiliations:** 1 Primary Care and Population Sciences, Faculty of Medicine, University of Southampton, Aldermoor Health Centre, Southampton, UK

## Abstract

Quality of life (QoL) has a weak relationship with lung function (LF) impairment in COPD; some cope well despite poor LF, whereas others suffer disproportionate QoL impairment despite well-preserved LF. Adjuvant non-pharmacological interventions such as rehabilitation and psychological/behavioural support may help if acceptable and targeted appropriately, but they are under-used and sometimes declined by patients. This study aimed to explore and understand variations in experiences and coping strategies in patients with different severities of disease and disease-specific QoL. Thirty-four participants were purposively sampled across a spectrum of LF and QoL impairment, to cover a grid of sub-groups (‘very severe LF, good QoL’, moderate LF, poor QoL’ and so on). Semi-structured interviews, digitally recorded, were analysed by thematic analysis. Data saturation was achieved. Four themes emerged: symptom impact, coping strategies, coping challenges and support needs. Most of them described using multiple coping strategies, yet over half reported significant challenges coping with COPD, including psychological impact, non-acceptance of diagnosis and/or disease progression, effects of co-morbidities and inadequate self-management skills. Approximately half of the participants wanted further help, ideally non-pharmacological, across all LF impairment groups but mainly with lower QoL. Those with lower QoL additionally reported greater psychological distress and greater use of non-pharmacological support strategies where accessible. Patients who develop effective coping strategies have a better QoL independent of objective LF, whereas others cope poorly, are aware of this and report more use of non-pharmacological approaches. This study suggests that severely impaired QoL, irrelevant of lung function, is a powerful patient-centred indication to explore the positive benefits of psychological and behavioural support for distressed COPD patients.

## Introduction

COPD is a major cause of morbidity and mortality; it is currently the fifth leading cause of death in the UK and predicted to become the third leading cause of death worldwide by 2020.^1^ Most patients are managed in primary care,^2^ accounting for 4–10% of GP consultations in the UK.^3^ Treatment aims are to improve symptoms and health-related quality of life (HRQoL) while minimising further damage and complications, exacerbations and hospital admissions. It is a complex, multifaceted disease with both somatic and affective components. Anxiety and depression are common,^4^ and psychological co-morbidity is associated with poor outcomes including impaired functional status,^5,6^ HRQoL^7–9^ and worsened prognosis.^10^ However, psychological co-morbidity is frequently unrecognised and untreated,^11,12^ which has potential implications for treatment compliance and health system outcomes such as hospital admissions and primary care consultation rates.^6,11,13^

As an incurable chronic disease, improving HRQoL is a major aim of COPD management.^14–21^ Prognosis has been shown to be related to HRQoL.^22,23^ The UK Department of Health Strategy for COPD^24^ highlights the need to recognise and address HRQoL impairment and psychological co-morbidity. However, because of a lack of clinical trials, there is currently a paucity of evidence on the most effective strategies to identify and address psychological co-morbidity in COPD, or on targeting these interventions to specific patient groups.

The relationship between physiological disease impairment (as measured by percentage predicted forced expiratory volume in the first second, FEV_1_) and patients’ disease experience^21^ is weak. Some patients have highly impaired HRQoL despite relatively minor lung function impairment, and others have good HRQoL despite severe lung function impairment. It is likely that psychological and behavioural factors may be relevant,^25–27^ but the coping strategies used by patients and their relationship to individual psychological factors have been incompletely explored. This qualitative study aimed to explore and understand variations in experiences and coping strategies in COPD patients across different severities of disease and disease impact.

## Results

### Participant characteristics

**Figure 1:**
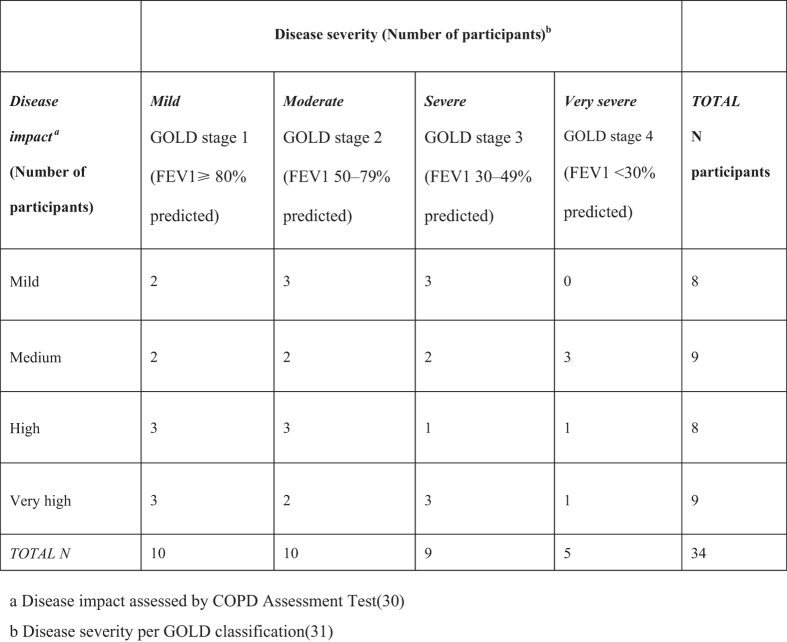
Participants’ recruitment sampling grid.

Thirty-four participants were interviewed across the differing severities of disease severity and HRQoL (Figure 1). Table 1 reports their characteristics. The mean interview length was 110 min (range 55–150 min); three interviews were terminated early because of poor health (breathlessness and/or fatigue).

### Findings

Four themes were identified. Sub-analysis exploring subgroup differences (between and within categories of disease severity and disease impact) are reported in the text at the end of each theme; Figure 1 highlights the key subgroup differences identified. Further details are reported in Supplementary Appendix 1.

## Theme 1: symptoms and impact

All but two participants described breathlessness, with other common symptoms being chest infections, cough, wheeze, fatigue and sleep disturbance. Those with more physiologically severe disease reported more frequent exacerbations and hospitalisation. Disease impact varied considerably, ranging from minimal or variable to severe impact, with major restrictions to daily life, with some of them requiring assistance with personal care and mobility aids. As dictated by the sampling matrix, the data confirmed a high degree of variability in COPD impact both within and between physiological severities. Although generally those with worse lung function tended to report more severe symptoms and impact, a high level of variability was noted, with some reporting low impact and active lives despite severely damaged lungs and others reporting severe impact despite well-preserved lung function. For example, two participants having the same GOLD classification (stage 4, very severe) reported experiencing completely different lives. Participant 17 explained how COPD significantly curtailed basic daily activities such as dressing,

*I’m quite sedentary. But I find just getting up in the morning and going to the bathroom, go to the toilet, have a wash, comb my hair, have a shower or whatever, clean my teeth, I have to go back and sit on the bed and get my breath back just from doing that. Just from putting my shoes on in the morning, even that can make me breathless.[ID17]*

whereas Participant 31 was enjoying active hobbies of sailing and dog walking.

*I still go sailing every week. I’ve still got a boat to play with... It just gets harder! ….every day normally, just walking the dogs and messing about like I do. Don’t forget in between time I’ve got to cook for myself as well. [ID31]*

In contrast, some participants with milder disease reported COPD as significantly affecting their life.

*Yes, terrible breathing problems. I can’t walk any distance without getting out of breath... I have a buggy for when I shop, but I’m tempted to get one of those wheeler things so as I can get my exercise, because I just sit and sit and sit, you know. It’s silly… because I can’t breathe I can’t do the exercise and I can’t do nothing [ID27]*

## Theme 2: coping strategies

A range of coping strategies were used, with most participants using more than one approach.

### Medication

Nearly all participants used prescribed medication for COPD. Most of them considered their medication effective, but four participants found their inhalers ineffective because of the difficulty in using them when breathless. Eight participants managed COPD with medication alone.

### Non-pharmacological coping strategies

Twenty-six participants reported using a variety of additional coping strategies. Ten had attended pulmonary rehabilitation (PR), and they reported that the information, exercises and breathing training were helpful.

*I’ve been on a COPD course for exercising with physiotherapists, there was a group of us, we could all chat together with our different problems and how we dealt with it. That was brilliant. That was a six week course. We were all sorry it ended. It was very good. [ID14]*

Fourteen participants reported exercising regularly, either continuing PR exercises or using other forms of exercise—e.g., walking daily. All perceived regular exercise helped them maintain their well-being or reduced medication reliance. Breathing exercises learnt either at PR, yoga or relaxation classes, swimming or singing lessons were also helpful.

*If I get stressed I try and control it [breathlessness]. I’ll say, ’Right, just sit down and think about this. What’s happening here? You’re breathing in’, [inhales loudly] and I do the three breaths. It sort of comes back to you. [ID29]*

Other participants used complementary and alternative medicine such as Reiki, osteopathy and salt pipes, and some of them also explored psychological approaches by visiting therapists managing depression/anxiety (hypnotherapy, cognitive behavioural therapy, relaxation and counselling), with varying effectiveness. Activity avoidance and limiting exercise to reduce dyspnoea was used by some.

*I have to think what I’m going to do and how much breath I’m going to need to do it, so I’m permanently thinking about it…. else I could end up in trouble,[ID28]*

Others made practical adjustments including the use of mobility aids, stair lifts and assistance with personal care and/or housework. A single participant attended a Breathe Easy (British Lung Foundation) patient support group.

**Figure 2:**
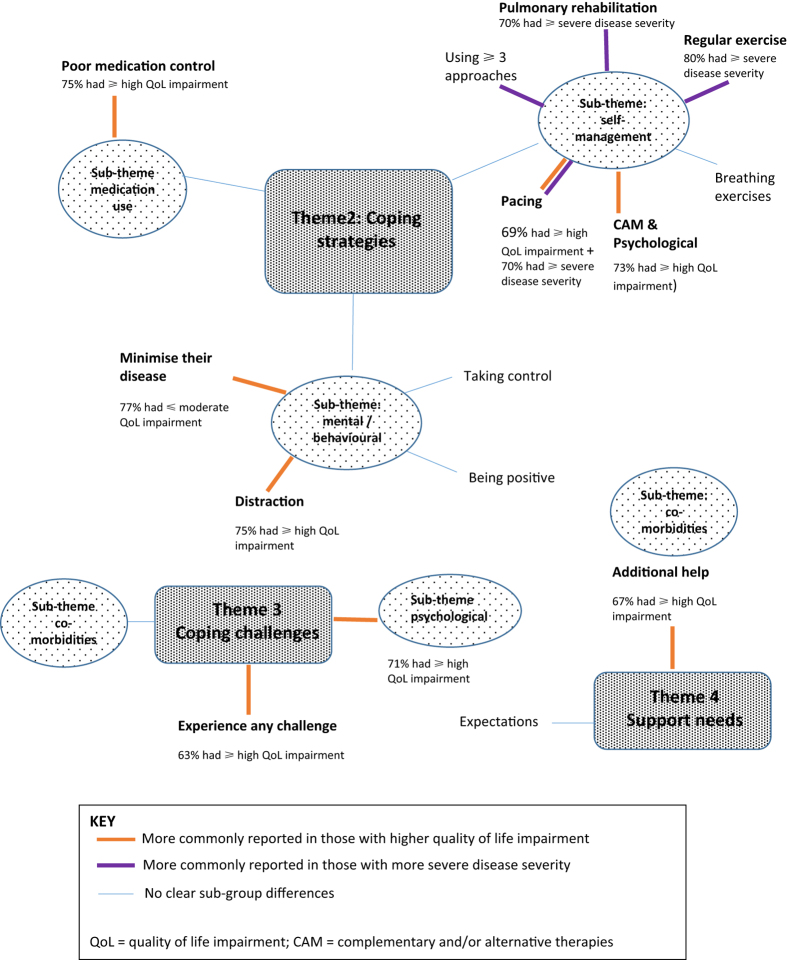
Diagrammatic representation of subgroup analysis.

Some subgroup differences were identified predicting multiple or specific types of strategies (Figure 2). Although most of the participants used more than one modality, seven participants were using three or more approaches, principally those with more severe physiological disease. Those with greater than equal to severe disease were more likely to have attended PR, to exercise regularly exercise and to use pacing. Those with greater than equal to high quality of life (QoL) impairment were generally more likely to seek complementary and alternative medicine or psychological treatments.

### Mental/behavioural coping strategies

Nearly all participants described one or more mental or behavioural coping strategies. ‘Taking control of COPD’ was an effective mechanism for those who could achieve it. These patients were determined that COPD would not control their life, and they continued an active life with hobbies and interests despite physical limitations.

*I try and do as much as I can and what I can—while I can; I will not let it defeat me... No way. I want to be able to, you know, keep my quality of life going …. that’s my motto. [ID3]*

Ways of enabling taking control included accepting their diagnosis and consequent limitations, having a realistic disease understanding and cultivating a rational, rather than an emotional, response to the disease;

*I already have got my own controls with it and I never get anxious…. It’s better to stay calm and address the situation and quantify it and go along with the way that you think you should.. [ID11]*

Other coping strategies reported also included maintaining emotional positivity despite symptoms,

*I’ve got a good mindset and I don’t let anything get me down really….generally speaking every day is a bonus for everybody really and I try and make the most of it. I have a car so I get out and then I can do my walking…[ ID11]*

as well as the use of ‘distraction’ and stress avoidance

*I just try and take my mind off it by reading. I might watch some telly. …. listening to my music. Just things to try and take my mind of it... Yes, that’s my way of coping with it really. [ID14]*

Finally, ‘minimising’ their disease by refusing to perceive themselves as ill, or by viewing the impact of COPD as relatively minor in comparison with other illnesses, was used by some.

*My inability to walk [because of a spinal condition] affects me a lot… it starts getting painful after about 50 yards and after 200 yards I really need to sit down...[the COPD] doesn’t affect me. Most of the time, I mean I should think probably two weeks a year I’m conscious of it.[ID5]*

Subgroup analysis identified that using a ‘minimising’ strategy was associated with lower QoL impairment, whereas those who used distraction to cope generally had highly impaired QoL (Figure 2).

## Theme 3: coping challenges

Nineteen participants were still struggling to cope with COPD either intermittently or persistently (Figure 2). Although most participants had co-morbidity, eleven reported that having multiple conditions was challenging.

*Yes, the coughing, breathlessness, it affects my diabetes,...diabetes causes infections and then they live off infections, the sugar, and so I do tend to get a lot of infections [ID14]*

Being depressed and/or anxious was also common, with six receiving treatment. Most of the participants attributed COPD as a cause and/or trigger for their depression/anxiety, and many of these had high HRQoL impairment relative to lung function. Anxiety, reported by thirteen participants, was associated with breathlessness.

*I’ve choked a few times, and I know what it’s like, and it’s quite frightening. So, yes, it is one of my biggest fears, is not being able to breathe. [ID13]*

For some participants, their anxiety significantly restricted their lives, with three reporting severe anxiety, panic attacks and agoraphobia.

*As I started to get older I started to get worse and the phlegm started to build up on my chest... you have some very dodgy moments through the course of the day. And, that’s when I started to really go downhill because I couldn’t breathe…it put me in [a] fear state really because I didn’t want to leave my nebuliser, because the nebuliser was the only thing that brought me round.... I had to have that nebuliser.., I had … about three years basically, really bad years...I didn’t go out. [ID34]*

Twelve participants reported being depressed, of whom five participants were taking antidepressant medication, two referred for psychological therapies and another two had independently tried meditation. Depression was viewed as a response to the limitations imposed by COPD.

*Well it [COPD] stops me doing things. It stops me gardening and it bothers me that way. I get frustrated with not being able to do things..... I’ve adapted to it now.. I don’t do anything full stop.. [ID 32]*

Some were still unable to accept their diagnosis.

*I still don’t accept it…it’s accepting, and accepting and moving on from it. Because that’s what causes, it’s the main factor in the depression. It is accepting and knowing my limitations. Helping, somebody to help me to accept that that’s what’s happening... I know the cough’s there. I know I’ve got COPD, but that doesn’t mean to say I’m going to accept it.[ID10]*

Others reported a sense of loss of wellness, a struggle to adjust and a feeling of being a burden on friends, family and doctors.

*I decided to take early retirement. So I retired just before.. I was 64. And, [pause] you know the general idea was to do a bit of travelling... But it’s restricted all that. So, all my dreams of what I was going to do faded away basically because of my lungs [ID34]*

Perceived lack of medical support also made coping challenging. Some felt stigmatised and blamed for having a smoking-related disease. Deteriorating disease was additionally challenging practically and emotionally.

*It’s at the stage where they can do nothing for me now. The last appointment I had with the specialist they wrote me off. … they won’t contact me anymore because there’s nothing else they can do. It’s a case of right that’s it, yeah, on your bike. [ID32]*

Participants who perceived COPD as particularly challenging had a range of physiological severities but generally had lower QoL and included most of those self-reporting depression and/or anxiety (Figure 2).

## Theme 4: support needs

Over half of them expressed a need and a desire for additional help with their breathing. Most of the participants understood the progressive nature of COPD, but had differing expectations about what further help could achieve. Some aspired to a cure despite recognising this was unrealistic.

*I just want them to find something, do something about it and get me back to normal. That’s all I want. [ID20]*

Although some did not expect anything could help, most of them wanted interventions to help their breathing, particularly those participants with greater psychological disturbance and impaired QoL. Many participants wanted symptom improvement, although most of them were reluctant to take further medication, generally expressing a preference for psychological support

*For me it’s acceptance. It’s not the cough. It’s not anything, it’s accepting, and accepting and moving on from it. Because that’s what causes, it’s the main factor in the depression.[ID10]*

or non-pharmacological treatments.

*I take enough [tablets] as it is, I don’t want any more. [ID6]*

The majority of those desiring further help had high QoL impact (Figure 2), and nearly all participants wanting help were also those reporting significant psychological impact from COPD.

## Discussion

### Main findings

In this qualitative study, we specifically used a stratified sampling strategy to investigate the variation in individual experience and range of coping strategies used by patients with differing degrees of physiological lung damage and quality-of-life impairment. This novel approach ensured that we collected data from people with the same level of lung function but with varying experience of QoL in order to make qualitative comparisons to gain insight into what personal and interventional strategies are helpful to patients. Most of our participants were using medication, and they often used multiple coping strategies either self-directed or supported by external interventions, such as PR. Yet for some, significant symptoms and lifestyle impact remained. Our key finding is that roughly half of the sample seemed to have managed to become relatively well adjusted to their on-going health impairment and were ‘making the best’ of life within the constraints of their condition. These individuals had adopted positive coping strategies that supported them in their desire to get on with an active and involved life. Unfortunately, the other half of our sample were having a much poorer QoL, which seemed to be driven by maladaptation to their on-going disease and the consequent behavioural, psychological and emotional dysfunction. Their coping strategies, such as activity limitation by ‘pacing’, distraction and using psychological therapies and complementary and alternative medicine appear to be less effective, and this group was characterised by much greater reporting of COPD-associated anxiety and depression. This group was aware that things were not going well for them, and conceded they needed help. They appeared willing to engage in interventions that could potentially help them further, particularly non-pharmacological interventions such as psychological and behavioural support.

### Interpretation of findings in relation to previously published work

Mirroring previous literature, we identified similar strategies that the participants used in coping with living with COPD, namely the use of non-pharmacological interventions including complementary and alternative medicine^28,29^ and psychological approaches, as well as mental and behavioural strategies such as pacing. Similarly, we also identified previously documented challenges impairing coping^30^ including managing co-morbidities,^30,31^ lacking self-management skills,^32^ depression and anxiety.^31–34^ Although quantitative data on the disparity between physiological severity and QoL have previously been reported,^21^ this novel study provides the first depth exploration of patient disease experiences underpinning this observation and includes a characterisation of a subgroup of patients with unmet needs. Our data suggest that the in/effectiveness of coping strategies may in part mediate their QoL, but this domain is not currently addressed in HRQoL measures.^35^

### Strengths and limitations of this study

There were some limitations to this study. Participants were purposively recruited using a maximum variation sample across physiological severities and HRQoL impairment for a wider study on perspectives of a non-pharmacological intervention; as such, recruited participants may have been more engaged than the broader community. We had some difficulty recruiting patients with the most severe LF impairment. The strengths were that data saturation was achieved, including for all subgroup analysis including very severe LF. A range of standard approaches to minimise bias were used, including a detailed audit trail, undertaking negative and deviant case analysis, cross-checking of coding strategies and a multi-disciplinary research team including Patient and Public Involvement input from COPD patients.

### Implications for future research, policy and practice

This qualitative hypothesis-generating study has identified potential future research questions. We feel that targeted interventional studies designed to improve self-management and coping strategies using non-pharmacological approaches for appropriately characterised patients are needed. There is currently an inadequate evidence base for most non-pharmacological interventions (other than PR) in COPD,^36,37^ and in particular for psychological and behavioural interventions. Our data suggest that such studies are likely to be attractive and may plausibly be effective in an identifiable subgroup of patients who appear willing to consider adopting them. This group can be identified by those with poor QoL, as assessed by a disease-specific health status instrument, regardless of physiological severity, who also self-report COPD-related depression and/or anxiety. We feel that this characterisation identifies a COPD ‘phenotype’ that should be targeted by such studies, rather than groups defined by the lung function (FEV_1_) or even breathlessness (MRC dyspnoea scale^38^) criteria that are currently used to stratify patients for interventions—e.g., PR programmes.

### Conclusions

In summary, our study provides a novel perspective on patients’ experience of living and coping with COPD. The data suggest that the mismatch previously described between QoL and objective LF in COPD may be mediated, at least in part, by variations in the effectiveness of the individual’s personal coping and self-management strategies, which can negatively have an impact on the QoL. This group, who have the most to gain from psychological and behavioural support interventions, are also likely to be receptive to such interventions.

This study suggests that severely impaired QoL, irrespective of lung function, is a powerful patient-centred indication to explore the positive benefits of psychological and behavioural support for distressed COPD patients.

## Materials and methods

### Design

This is a qualitative study of individual face-to-face semi-structured interviews analysed by thematic analysis.^39,40^

### Sampling and recruitment

Patients with a physician-confirmed COPD and quality-assured spirometry readings (to permit physiological impairment categorisation) and COPD disease-specific QoL assessments using the validated COPD Assessment Test, CAT,^41^ were recruited from primary and secondary care sites and a patient volunteer database in South England. Participants were purposively sampled to constitute a maximum variation sample across all severities of physiological disease (mild to very severe lung function impairment using GOLD classification^42^) and disease-specific QoL (low to very high disease impact using the CAT); Figure 1. Potential participants were identified on the basis of physiological disease severity (recent percentage predicted FEV_1_) and screened by telephone using CAT.^41^ Those fitting the planned sampling matrix were invited to interview. A total of 729 people with COPD were approached, 136 (18.4%) responded and were telephone screened, 36 were recruited and 34 were interviewed (2 dropped out for health reasons).

### Data collection

Interviews were conducted by SB (from December 2013 to April 2014) generally in the participants’ home. Written informed consent was obtained before interview. The topic guide explored the following: experience of symptoms and perceived impact; managing COPD; challenges; and perceived need for additional support. Interviews were conducted until data saturation was achieved. Interviews were digitally recorded and transcribed verbatim for textual analysis using pseudonyms. Reflexive notes, completed after each interview, aided analysis. Ethical approval was obtained from the East Midlands Research Ethics Committee (13/EM/0315) and sponsorship from the host institution.

### Data analysis

Interviews were imported into Atlas.ti v5.2 (ATLAS.ti Scientific Software Development GmbH, Berlin, Germany) for coding and thematic analysis.^39,40^ Analysis was conducted both within and across individual interviews to identify group similarities and differences including negative and deviant case analysis. Interviews were analysed line by line using open coding and then grouped into themes and subthemes encapsulating all participants’ experiences. Subgroup analysis was conducted to explore any differences across severities of disease severity and QoL impairment. Analysis confirmed that saturation was achieved (including exploring subgroup differences), with no new findings identified after 32 interviews. Illustrative quotations from the interviews describing the themes and subthemes are reported in the text.

## Funding

The study was funded by NIHR School for Primary Care Research (reference 195).

## Figures and Tables

**Table 1:** Participants’ characteristics

*Characteristics*	*Numbers of participants*
Gender (male: female)	21 male: 13 female
Age (years) (mean: range)	72.2: 39–86
Years since diagnosis (years) (mean: range)	9.4: 0.5–49

*Severity of COPD (GOLD)*^42^	
GOLD stage 1 (FEV_1_⩾80% predicted)	10
GOLD stage 2 (FEV_1_ 50–79% predicted)	10
GOLD stage 3 (FEV_1_ 30–49% predicted)	9
GOLD stage 4 (FEV_1_<30% predicted, or <50% predicted with chronic respiratory failure present)	5
	
*Health-related quality-of-life impact (COPD Assessment Test, CAT^41^)*	
Low (CAT Score<10)	8
Medium (CAT Score 10–20)	9
High (CAT Score 20–30)	8
Very high (CAT Score>30)	9
Previous attendance at pulmonary rehabilitation	10
	
*Living conditions*	
Alone: with spouse/family	11: 23
Accommodation type	28 own home: 5 sheltered housing: 1 hostel
Working status	Unemployed: 1
	Part-time working: 2
	Semi-retired: 2
	Retired: 29

*Co-morbidities*	
Number of participants with co-morbidity	32
Mean number: range	3.4: 0–11
*Self-reported psychological co-morbidities* (Numbers: receiving treatment (medication/psychological therapies)	
Any	19: 6
Anxiety	13: 3
Depression	12: 5
Anxiety and depression	8: 3

Abbreviations: COPD, chronic obstructive pulmonary lung diseae; FEV_1_, forced expiratory volume in the first second; GOLD, Global Initiative for Chronic Obstructive Lung Disease.

## Notes

MT is an Associate Editor of npj Primary Care Respiratory Medicine, but was not involved in the editorial review of, nor the decision to publish, this article. The authors declare no conflict of interest.
