# Supplementary material for: Patient coping strategies in COPD across disease severity and quality of life: a qualitative study
Source: NPJ Prim Care Respir Med. 2016 Sep 15;26:16051–. doi: 10.1038/npjpcrm.2016.51 (PMC5024412; doi:10.1038/npjpcrm.2016.51)
Supplement: Supplementary Information [file npjpcrm201651-s1.doc]

**Supplementary Information**

**~~Table 2~~ Appendix 1. Sub-analysis ~~for~~ across severities of disease severity and HRQoL impairment for Themes 2 to 4**

| **THEME** | **SUB-THEME** | | **N reporting** | **Differences across Disease severity^a^**  (% of sample using approach) | **Differences across QoL impairment^b^**  (% of sample using approach) |
| --- | --- | --- | --- | --- | --- |
| **MANAGING COPD** | **Medication use** | Poor medication control | 4 | - | 75% ≥high QoL impairment |
|  | **Self-management** | PR | 10 | 70% ≥ severe disease severity | - |
|  |  | Regular exercise | 14 | 80% ≥ severe disease severity | - |
|  |  | Breathing exercise | 12 | - | - |
|  |  | Complementary /alternative therapies & psychological therapies | 11 |  | 73% ≥ high QoL impairment |
|  |  | Pacing | 13 | 70% ≥ severe disease severity | 69% ≥high QoL impairment |
|  |  | N using ≥3 of these approaches | 7 | 71% ≥ severe |  |
|  | **Mental coping** | Taking control | 13 | - | - |
|  |  | Being positive | 12 | - | - |
|  |  | Minimise disease | 13 |  | 77%≤moderate QoL impairment |
|  |  | Distraction | 4 |  | 75% ≥high QoL impairment |
| **CHALLENGES COPING** | Experiencing any challenge | ~~Experiencing any challenge~~ | 19 | - | 63% ≥high QoL impairment |
|  | Psychological | ~~Psychological~~ | 17 | - | 71% ≥high QoL impairment |
|  | Comorbidities | ~~Comorbidities~~ | 11 | - | - |
| **~~WANTS ADDITIONAL HELP~~**  **SUPPORT NEEDS** |  |  | 18 | - | 67% ≥high QoL impairment |

1. Sub group analysis across disease severity was determined by comparing those with milder disease severity (GOLD stage 1+2) versus those with more severe disease severity (GOLD stage 3+4)(31)
2. Sub group analysis across QoL impairment was determined by those with higher HRQoL (i.e. those reporting mild and medium categories on the COPD Assessment Test) versus those with lower HRQoL ~~severe disease~~ severity (i.e. those reporting high and very high categories on the COPD Assessment Test )(30)

(-)= no clear sub-group differences
